# Morphological and phylogenetic evidence reveal two new *Pseudohydnum* (*Auriculariales*, *Basidiomycota*) species from Guangxi and Xizang, China

**DOI:** 10.3897/mycokeys.131.189424

**Published:** 2026-04-24

**Authors:** Xin Zhang, Hong-Min Zhou, Zhe-Hui Tu, Guang-Yu Zeng, Yu-Cheng Dai

**Affiliations:** 1 School of Ecology and Nature Conservation, Beijing Forestry University, Beijing 100083, China College of Forestry, Southwest Forestry University Kunming China; 2 College of Forestry, Southwest Forestry University, Kunming 650224, China School of Ecology and Nature Conservation, Beijing Forestry University Beijing China; 3 Guangxi Forestry Research Institute, Nanning 530002, Guangxi, China Guangxi Forestry Research Institute Nanning China

**Keywords:** *
Auriculariales
*, China, jelly fungi, new species, phylogeny, taxonomy

## Abstract

*Pseudohydnum (Auriculariales)* is characterized by gelatinous, hydnoid basidiomata and longitudinally septate basidia. Currently, 26 species and one subspecies are accepted worldwide with molecular evidence, including two new species described here. In this study, specimens from Guangxi, Xizang, and Jilin provinces, China, were examined using morphological and phylogenetic analyses based on ITS1–5.8S–ITS2–nLSU sequences. The phylogeny resolves the genus as a well-supported monophyletic group. *Pseudohydnum umbrosum* is newly recorded from China. Two new species are described and illustrated as *P. fusiformis* from Xizang, characterized by olivaceous buff to clay-pink basidiomata, association with *Abies* at high elevations (above 3200 m), and the presence of fusiform cystidia—a feature previously known only from *P. cystidiatum* within the genus; and *P. rhododendri* from Guangxi, characterized by shell-shaped basidiomata with a pale mouse-gray pileal surface, the presence of cystidioles, and growing on living *Rhododendron*. The discovery of these new taxa and the new record further highlight China as a significant diversity center for *Pseudohydnum*.

## Introduction

*Pseudohydnum* P. Karst. was established by Karsten with *P. gelatinosum* (Scop.) P. Karst. as the type species (Karsten 1868). The genus is characterized by gelatinous, laterally stipitate basidiomata with a hydnoid hymenophore, longitudinally septate phragmobasidia, and smooth, subglobose to ellipsoid basidiospores (Karsten 1868; Lowy 1971; Chen et al. 2020; Zhou et al. 2022, 2023). These distinctive features easily distinguish *Pseudohydnum* from other genera in *Auriculariales*, such as *Auricularia* Bull. (gelatinous, resupinate to substipitate basidiomata with hairs, transversely septate basidia); *Eichleriella* Bres. (leathery to ceraceous, cupulate to resupinate basidiomata with a smooth hymenophore); and *Exidia* Fr. (gelatinous basidiomata, allantoid basidiospores; Malysheva and Spirin 2017; Spirin et al. 2019a; Wu et al. 2021).

The genus was traditionally addressed in *Tremellales* due to its longitudinally septate basidia (Karsten 1868; Breitenbach and Kränzlin 1986). However, Bandoni (1984) redefined the concept of *Auriculariales* based on micromorphological, ultrastructural, ecological, and developmental data and suggested that *Pseudohydnum* belongs to this order. Subsequent phylogenetic studies have confirmed this placement, although the exact position of the genus within *Auriculariales* remains ambiguous (Weiß and Oberwinkler 2001; Malysheva and Spirin 2017; Spirin et al. 2019c, 2025). The presence of hydnoid basidiomata in *Pseudohydnum* indicates a distinctive classification status within *Auriculariales*, as taxa with diverse basidiomata morphologies, including jelly, stereoid, corticioid, hydnoid, and poroid forms, are not typically grouped together in the same order (Malysheva and Spirin 2017; Spirin et al. 2019c; Wu et al. 2021).

Recent taxonomic revisions and extensive field collections have substantially increased knowledge on the diversity of *Pseudohydnum*. Currently, 25 taxa, including 24 species and one subspecies with molecular evidence, are accepted in the genus, distributed across Asia, Europe, North America, South America, and Oceania, with no representatives yet documented from Africa (Karsten 1868; Breitenbach and Kränzlin 1986; Niveiro and Popoff 2011; Chen et al. 2020; Zhou et al. 2022, 2023; Spirin et al. 2023; Coelho-Nascimento et al. 2024; Lei et al. 2025; Wang et al. 2025b; Spirin et al. 2025). China, with its diverse forest ecosystems, is recognized as a significant biodiversity hotspot for macrofungi (Dai et al. 2010; Chen et al. 2015; Wu et al. 2019; Wang et al. 2023, 2024, 2025a, 2025b; Dong et al. 2024; Liu et al. 2025; Song et al. 2025; Yang et al. 2025; Zhao et al. 2025). Among them, 18 species have been recorded from Asia, including 17 species from China, highlighting the region as a significant diversity center for the genus. These Asian species are *P. abietinum* H.M. Zhou & Jing Si (Gansu of China), *P. brunneiceps* Y.L. Chen et al. (Jiangxi of China), *P. cystidiatum* Malysheva & V. Dudka (Yunnan of China, Vietnam), *P. himalayanum* Y.C. Dai et al. (Yunnan, Xizang of China), *P. laricicola* Zhu L. Yang et al. (Sichuan of China), *P. meridianum* Malysheva & Spirin (Yunnan of China, Vietnam), *P. motuoense* L. Lei & Q. Zhao (Xizang of China), *P. placibile* Malysheva & V. Dudka (Vietnam), *P. purum* L. Lei & Q. Zhao (Xizang of China), *P. sinobisporum* T. Bau et al. (Jilin, Heilongjiang of China), *P. sinogelatinosum* Y.C. Dai et al. (Yunnan, Sichuan, Xizang of China), *P. sororium* Zhu L. Yang & Q. Cai (Yunnan of China), *P. translucens* Lloyd (Jilin of China, Japan, Siberia, Russia Far East), and *P. umbrosum* Malysheva & Spirin (Russia Far East, also reported from Jilin of China in this study), as well as the type species *P. gelatinosum* (Scop.) P. Karst. (Inner Mongolia of China, Chen et al. 2020; Zhou et al. 2022, 2023; Spirin et al. 2023; Lei et al. 2025; Wang et al. 2025b). Notably, *P. gelatinosum* occurs in both Europe and Asia, representing a widespread Eurasian taxon, whereas *P. alienum* Spirin & Malysheva is so far found in Europe and the Caucasus region (Finland, Georgia, and the Russian Caucasus; Spirin et al. 2023).

The type species, *Pseudohydnum gelatinosum*, originally described from Croatia, has a wide distribution in Europe (Karsten 1868; Wojewoda 1981) and has also been reported from Asia (China) and North America (Zhou et al. 2022; Spirin et al. 2023). In the Caribbean, the species has been recorded from Jamaica, Puerto Rico, and recently from Cuba (Lowy 1971; Minter et al. 2001; Castro Hernández et al. 2022). In North America, in addition to *P. gelatinosum* s. str., one subspecies, *P. gelatinosum* subsp. *pusillum* (Ellis & Everh.) Miettinen & Viner, and another species, *P. omnipavum* Spirin & Miettinen, have been documented (Spirin et al. 2023). South America, particularly the Brazilian Atlantic Forest, harbors four endemic species: *P. brasiliense* C.C. Nascim. et al., *P. brunneovelutinum* C.C. Nascim. & Menolli, *P. cupulisnymphae* C.C. Nascim. & Menolli, and *P. viridimontanum* C.C. Nascim. & Menolli (Coelho-Nascimento et al. 2024). Oceania harbors three endemic species: *P. orbiculare* J.A. Cooper, *P. tasmanicum* Y.C. Dai & G.M. Gates, and *P. totarae* (Lloyd) J.A. Cooper (Zhou et al. 2022).

While studying specimens of jelly fungi collected from China, several interesting taxa were identified. Among them, *Pseudohydnum umbrosum*, previously described from the Russian Far East, was discovered as a new record for the country. Additionally, two unknown taxa, morphologically distinct from all previously described species, were collected from Guangxi and Xizang provinces. In this study, phylogenetic analyses were performed based on the internal transcribed spacer (ITS1–5.8S–ITS2) and large subunit nuclear ribosomal RNA gene (nLSU) sequences to determine their phylogenetic positions. Combining morphological and molecular evidence, two new species, *Pseudohydnum fusiformis* from Xizang and *P. rhododendri* from Guangxi, are described and illustrated.

## Materials and methods

### Morphological studies

The specimens used in this study are deposited at the Fungarium of the State Key Laboratory of Efficient Production of Forest Resources, Beijing Forestry University, China (**BJFC**), and the Herbarium of Cryptogams at the Kunming Institute of Botany, Chinese Academy of Sciences, China (**KUN-HKAS**). Samples were photographed *in situ*, and important macro-morphological features were recorded. Morphological studies followed Wang et al. (2025a) and Zhou et al. (2022, 2023). Color terms follow Anonymous (1969) and Petersen (1996). Hand-cut sections of basidiomata were first treated with 5% KOH for a few minutes and then with 1% phloxine B (C_20_H_4_Br_4_Cl_2_K_2_O_5_). Micromorphological characters were examined with a Nikon Digital Sight DS-L3 or Leica ICC50 HD camera at magnifications up to 1000 times. At least 30 basidiospores per specimen were measured. For presentation of the size variation, 5% of the measurements were excluded from each end of the range and are provided in parentheses. Stalks were excluded for basidia measurement, and the hilar appendages were excluded for basidiospore measurement. The following abbreviations were used: **IKI** = Melzer’s reagent, **IKI−** = neither amyloid nor dextrinoid, **CB** = Cotton Blue, **CB−** = acyanophilous in Cotton Blue, ***L*** = arithmetic average length of all measured spores, ***W*** = arithmetic average of all measured spore widths, ***Q*** = *L*/*W* ratios among the studied specimens, and ***n* (*a*/*b*)** = number of spores (*a*) measured from a given number of specimens (*b*).

### Molecular studies and phylogenetic analysis

Genomic DNA was extracted from dried basidiomata using the CTAB plant genomic DNA extraction kit DN14 (Aidlab Biotechnologies Co., Ltd., Beijing, China) following the manufacturer’s guidelines with some modifications (Fan et al. 2021; Zhou et al. 2022). The ITS1–5.8S–ITS2 region was amplified using the primer pairs ITS4 and ITS5, and the nLSU region was amplified using the primer pairs LROR and LR7 (White et al. 1990; Hopple and Vilgalys 1994). The PCR procedures for the ITS1–5.8S–ITS2 and nLSU regions followed Zhou et al. (2023). All newly generated sequences were submitted to GenBank and are listed in Table 1.

**Table 1.** Taxa information and GenBank accession numbers of the sequences used in this study.

Species name	Samples	Locality	ITS no.	nLSU no.	References
*Protomerulius subreflexus*	OM 14402.1	Indonesia	MG757508	MG757508	Spirin et al. 2019b
*P. substuppeus*	O 19171	Costa Rica	JX134482	JQ764649	Spirin et al. 2019b
*Pseudohydnum abietinum*	Dai 24185*	China: Gansu	OP965350	OP965370	Zhou et al. 2023
*P. abietinum*	Dai 24194	China: Gansu	OP965351	OP965371	Zhou et al. 2023
*P. alienum*	Kotiranta 22407*	Finland	OM451494	OM451435	Spirin et al. 2023
*P. alienum*	LE 253853	Russia	OM451493	OM451430	Spirin et al. 2023
*P. brasiliense*	MPD570*	Brazil	OR625545	OR625561	Coelho-Nascimento et al. 2024
*P. brasiliense*	MPD572	Brazil	OR625546	OR625562	Coelho-Nascimento et al. 2024
*P. brunneiceps*	JXSB 0967*	China: Jiangxi	MN497254	MN497259	Chen et al. 2020
*P. brunneiceps*	JXSB0967-1	China: Jiangxi	MN497255	MN497260	Chen et al. 2020
*P. brunneovelutinum*	AGS118/2022	Brazil	OR625541	OR625557	Coelho-Nascimento et al. 2024
*P. brunneovelutinum*	NCC182	Brazil	OR625547	OR625563	Coelho-Nascimento et al. 2024
*P. cupulisnymphae*	NCC229	Brazil	OR625548	OR625564	Coelho-Nascimento et al. 2024
*P. cupulisnymphae*	NMJ420*	Brazil	OR625556	OR625570	Coelho-Nascimento et al. 2024
*P. cystidiatum*	HKAS 137494	China: Yunnan	PQ871457	PV276931	Wang et al. 2025b
*P. cystidiatum*	LE 313656*	Vietnam	OM451503	OM451463	Spirin et al. 2023
*P. cystidiatum*	LE 313657	Vietnam	OM451502	OM451464	Spirin et al. 2023
***P. fusiformis***	**Cui 12214**	**China: Xizang**	**PZ043114**	-	This study
***P. fusiformis***	Dai 23556*	**China: Xizang**	**PZ043115**	**PZ043120**	This study
***P. fusiformis***	**WHM5603**	**China: Xizang**	**PZ043116**	-	This study
*P. gelatinosum*	Dai 21665	China: Inner Mongolia	ON243826	ON243924	Zhou et al. 2022
*P. gelatinosum*	Spirin 13369	Slovenia	OM451472	OM451434	Spirin et al. 2023
*P. gelatinosum*	LE 313661	Russia	OM451469	OM451466	Spirin et al. 2023
*P. gelatinosum* ssp. *pusillum*	Miettinen 16894	USA	ON117832	-	Spirin et al. 2023
*P. gelatinosum* ssp. *pusillum*	Miettinen 18987.2*	USA	ON117833	-	Spirin et al. 2023
*P. gelatinosum* ssp. *pusillum*	Miettinen 19625.1	USA	ON117834	ON117839	Spirin et al. 2023
*P. himalayanum*	Cui 17045*	China: Yunnan	ON243829	ON243927	Zhou et al. 2022
*P. himalayanum*	Cui 17065	China: Yunnan	ON243830	ON243928	Zhou et al. 2022
*P. laricicola*	HKAS 103433*	China: Sichuan	PQ871449	PV650295	Wang et al. 2025b
*P. laricicola*	HKAS 103450	China: Sichuan	PQ871450	PV276933	Wang et al. 2025b
*P. meridianum*	HKAS 145328	China: Yunnan	PQ871458	PQ877881	Wang et al. 2025b
*P. meridianum*	HKAS 145329	China: Yunnan	PV650322	PQ877882	Wang et al. 2025b
*P. meridianum*	LE 313568*	Vietnam	OM451501	OM451452	Spirin et al. 2023
*P. motuoense*	HKAS 134347*	China: Xizang	PP999440	PP998493	Lei et al. 2025
*P. motuoense*	HKAS 134348	China: Xizang	PP999439	PP998492	Lei et al. 2025
*P. omnipavum*	Miettinen 18877	USA	ON117836	OM451460	Spirin et al. 2023
*P. omnipavum*	Spirin 8667*	USA	OM451499	OM451441	Spirin et al. 2023
*P. orbiculare*	PDD 112653*	New Zealand	ON243831	ON243929	Zhou et al. 2022
*P. orbiculare*	PDD 112654	New Zealand	ON243832	-	Zhou et al. 2022
*P. placibile*	LE 313658*	Vietnam	OM451505	OM451465	Spirin et al. 2023
*P. placibile*	LE 313659	Vietnam	OM451504	OM451462	Spirin et al. 2023
*P. purum*	HKAS 134352*	China: Xizang	PP999437	PP998490	Lei et al. 2025
*P. purum*	HKAS 134353	China: Xizang	PP999438	PP998491	Lei et al. 2025
***P. rhododendri***	Cui 14495*	**China: Guangxi**	**PZ043112**	**PZ043118**	This study
***P. rhododendri***	**Cui 14496**	**China: Guangxi**	**PZ043113**	**PZ043119**	This study
*P. sinobisporum*	HMJAU 33728	China: Heilongjiang	OP965349	OP965369	Zhou et al. 2023
*P. sinobisporum*	SYL 2307*	China: Jilin	OP965348	OP965368	Zhou et al. 2023
*P. sinogelatinosum*	Cui 17074	China: Yunnan	ON243834	ON243930	Zhou et al. 2022
*P. sinogelatinosum*	Dai 23017*	China: Yunnan	ON243836	ON243932	Zhou et al. 2022
*P. sororium*	HKAS 108870*	China: Yunnan	PQ871453	PQ877885	Wang et al. 2025b
*P. sororium*	HKAS 145330	China: Yunnan	PQ871454	PQ877886	Wang et al. 2025b
*P. sororium*	HKAS 145331	China: Yunnan	PQ871455	PQ877887	Wang et al. 2025b
*P. tasmanicum*	Cui 16721*	Australia	ON243838	ON243934	Zhou et al. 2022
*P. tasmanicum*	Dai 18724	Australia	ON243839	ON243935	Zhou et al. 2022
*P. totarae*	PDD 112652	New Zealand	ON243841	-	Zhou et al. 2022
*P. totarae*	PDD 112655	New Zealand	ON243842	ON243936	Zhou et al. 2022
*P. translucens*	Dai 23740	China: Jilin	OP965345	OP965365	Zhou et al. 2023
*P. translucens*	LE 313582	Russia	OM451498	OM451459	Spirin et al. 2023
*P. translucens*	LE 313586	Russia	OM451496	OM451455	Spirin et al. 2023
*P. umbrosum*	HMJAU6847	China: Jilin	**PZ043117**	**PZ043121**	This study
*P. umbrosum*	LE 312767*	Russia	OM451500	OM451453	Spirin et al. 2023
*P. viridimontanum*	NCC266*	Brazil	OR625552	OR957373	Coelho-Nascimento et al. 2024
*P. viridimontanum*	NCC273	Brazil	OR625553	OR957374	Coelho-Nascimento et al. 2024
*Stypellopsis farlowii*	GB Larsson 12337*	USA	NR_164269	MG857099	Spirin et al. 2019a
*S. hyperborea*	Spirin 11066 (O)	Norway	MG857096	MG857102	Spirin et al. 2019a

The new species and newly generated sequences are shown in bold. “*” indicating the holotype.

Sequences generated for this study and additional sequences downloaded from GenBank were partitioned into ITS1, 5.8S, ITS2, and nrLSU and then aligned separately using MAFFT v.74 (http://mafft.cbrc.jp/alignment/server/; Katoh et al. 2017) with the G-INS-i iterative refinement algorithm. Following manual optimization in BioEdit 7.0.5.3 (Hall 1999), the separate alignments were concatenated with Mesquite v.4.02 software (Maddison and Maddison 2025). The combined ITS1–5.8S–ITS2–nLSU dataset was analyzed to determine the phylogeny of *Pseudohydnum*, and sequences of *Protomerulius substuppeus* (Berk. & Cooke) Ryvarden and *Protomerulius subreflexus* (Lloyd) O. Miettinen & Ryvarden were used as outgroups following Zhou et al. (2022, 2023). The resulting alignment was deposited at TreeBase (submission ID 32526; Reviewer access URL: http://purl.org/phylo/treebase/phylows/study/TB2:S32526?x-access-code=70391994d471bfdc00e3cfdad510d1d5&format=html). Maximum likelihood (ML) and Bayesian inference (BI) methods were used for the phylogenetic analysis. ModelFinder v.2.2.0 with the corrected Akaike information criterion (AICc) was applied to estimate the best-fit partition scheme and evolutionary model for BI and ML analyses (Kalyaanamoorthy et al. 2017).

Maximum likelihood (ML) analysis was performed in RAxML v.8.2.12 (Stamatakis 2014) using the Cipres Science Gateway (http://www.phylo.org/portal2; Miller et al. 2010). All parameters in the ML analysis used default settings, and statistical support values were obtained using rapid bootstrapping with 1000 replicates.

Bayesian inference (BI) analysis was run with four chains for two independent runs and performed for two million generations, sampling every 1000 generations in MrBayes v.3.2.7 (Ronquist et al. 2012), until the split deviation frequency value was less than 0.01. The first 25% of the sampled trees were discarded as burn-in, and the remaining trees were used to reconstruct a majority-rule consensus and calculate BPP of the clades.

Trees were viewed in FigTree v.1.4.4 (http://tree.bio.ed.ac.uk/software/figtree/, Rambaut 2018). Branches that received bootstrap support for ML and Bayesian posterior probabilities (BPP) greater than or equal to 70% (ML) and 0.90 (BPP) were considered to be significantly supported, following previous studies (Zhou et al. 2022; Lei et al. 2025).

## Results

### BLAST analysis of ITS sequences for the new species

BLAST searches of the ITS sequences of the holotype specimens against the GenBank database showed that the sequence of *P. rhododendri* (Cui 14495) was most similar to the type sequence of *P. placibile* (NR_198211.1), with 89.98% identity (100% query cover). The sequence of *P. fusiformis* (Dai 23556) was most similar to *P. omnipavum* (OM451499.1), with 97.04% identity (100% query cover). These low to moderate similarity values (89.98–97.04%) support the recognition of the two isolates as distinct species from their closest relatives.

### Phylogeny of *Pseudohydnum*

**Figure 1.**
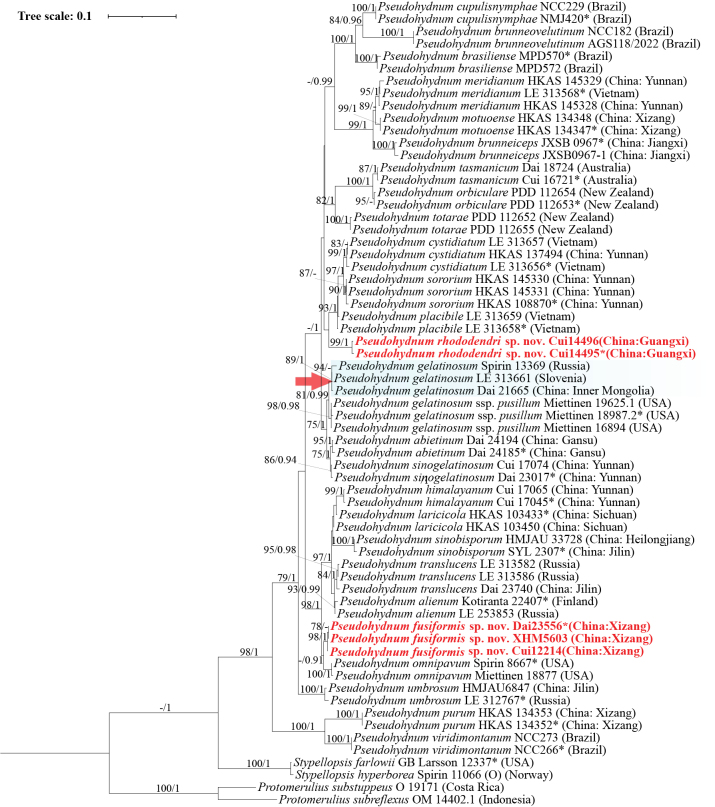
Maximum likelihood tree (ML) illustrating the phylogeny of *Pseudohydnum* based on a combined ITS1–5.8S–ITS2–nLSU dataset. Branches are labeled with ML bootstrap values (BS) higher than 70% and Bayesian posterior probabilities (BPP) higher than 0.90. New species are indicated in red bold with “sp. nov.”; the type species is indicated by a red arrow; type specimens are indicated with an asterisk (*).

The combined ITS1–5.8S–ITS2–nLSU dataset contained 61 ITS and 55 nrLSU sequences from 61 specimens, representing 27 taxa and the outgroup (Table 1). For Bayesian inference (BI), ModelFinder suggested SYM+G4 as the best-fit model for ITS1, 5.8S, and ITS2, and GTR+F+I+G4 as the best-fit model for nrLSU. For maximum likelihood (ML), the best-fit models were GTR+F+G4 for ITS1, 5.8S, and ITS2, and GTR+F+I+G4 for nrLSU. The BI analysis generated a topology nearly identical to that obtained from the ML analysis, with an average standard deviation of split frequencies of 0.006406. Accordingly, only the ML tree is presented (Fig. 1), with ML bootstrap values (BS) ≥ 70% and Bayesian posterior probabilities (BPP) ≥ 0.90 shown on branches.

The ITS1–5.8S–ITS2–nLSU-based phylogeny showed that *Pseudohydnum* is a well-supported monophyletic group comprising 27 taxa (98% ML, 1 BPP; Fig. 1). Two new species, described in this study as *Pseudohydnum fusiformis* and *Pseudohydnum rhododendri*, were nested within *Pseudohydnum* but occupied distinct phylogenetic positions.

Three specimens of *Pseudohydnum fusiformis* from Xizang, southwestern China, formed a distinct and strongly supported lineage (100% ML, 0.99 BPP) that formed a small subclade with *P. omnipavum* (0.91 BPP; Fig. 1). Together with a well-supported subclade (98% ML, 1 BPP) comprising *P. alienum*, *P. himalayanum*, *P. laricicola*, *P. sinobisporum*, and *P. translucens*, they formed a large well-supported clade (99% ML, 1 BPP).

Two specimens of *Pseudohydnum rhododendri* from Guangxi, southern China, formed an independent and strongly supported lineage (100% ML, 1 BPP) within *Pseudohydnum*.

### Taxonomy

#### Pseudohydnum fusiformis

Taxon classificationFungiAuricularialesHyaloriaceae

Xin Zhang & Y.C. Dai sp. nov.

5C3BBCC4-6617-5B44-B109-5AA19D5C4166

862555

Fig. 2

##### Diagnosis.

It is characterized by solitary basidiomata with a lateral stipe paler than pileal surface when fresh, the presence of fusiform cystidia, and growing on *Abies delavayi* var. *motuoensis* at high elevations (above 3200 m).

**Figure 2.**
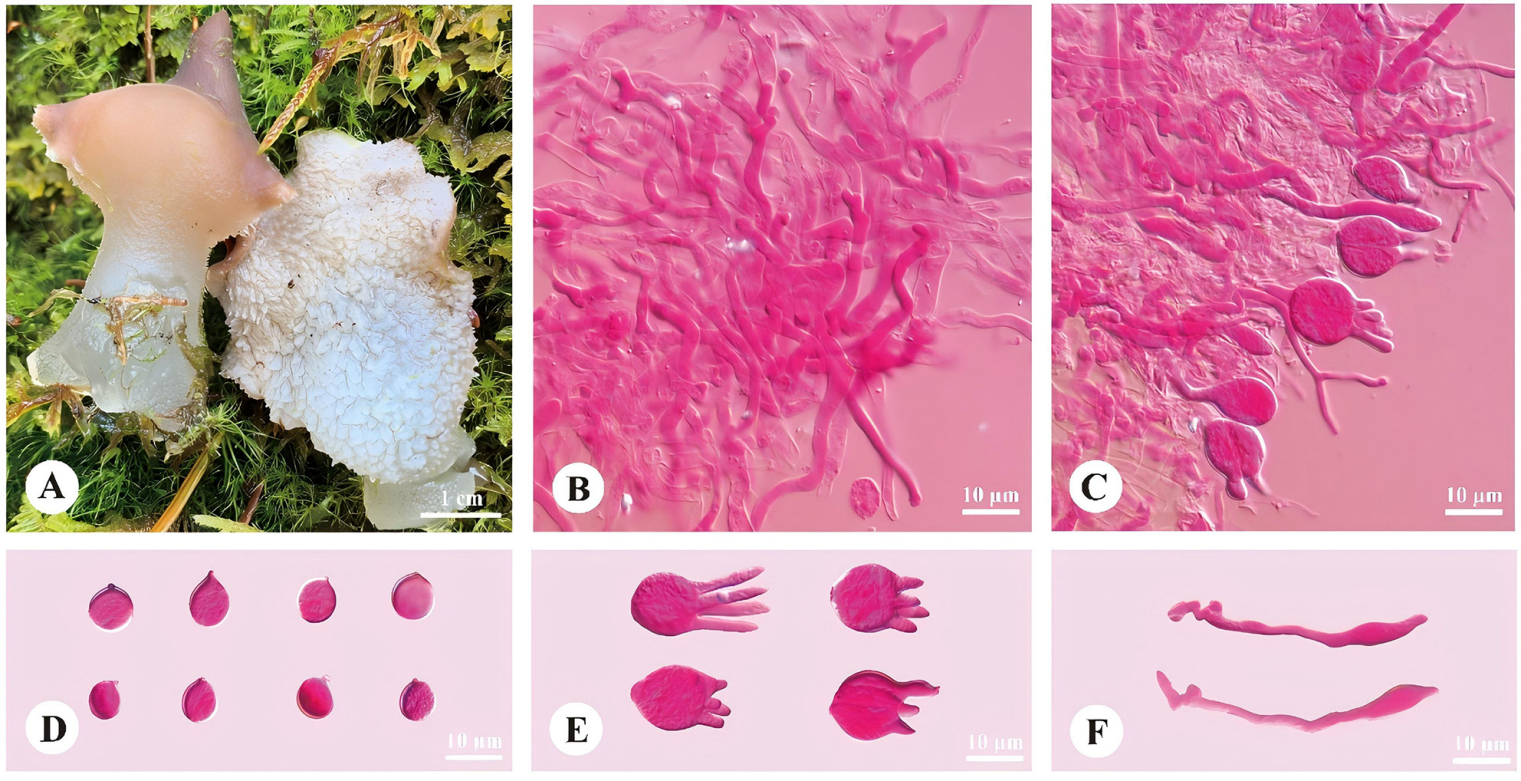
Basidiomata and microscopic structures of *Pseudohydnum fusiformis* (holotype, Dai 23556). **A**. Basidiomata; **B**. Tramal hyphae; **C**. A section of the hymenium; **D**. Basidiospores; **E**. Basidia; **F**. Cystidia.

##### Holotype.

China • Xizang Autonomous Region, Linzhi, Bomi County, the road from Bomi to Motuo, on fallen trunk of *Abies delavayi* var. *motuoensis*, elev. 3500 m, 26 October 2021, Dai 23556 (BJFC038128, holotype).

##### Etymology.

*Fusiformis* (Lat.): refers to the fusiform cystidia of the species.

##### Fruitbody.

Basidiomata annual, gelatinous when fresh, brittle when dry, solitary, with a lateral stipe. Pilei irregular, projecting up to 2.4 cm, 1.8 cm wide and 2.2 mm thick when dry. Pileal surface olivaceous buff (4C4) to clay-pink (6D4) when fresh, become cigar brown (6F5) when dry. Spines white (1A1), paler than pileal surface, conical when fresh, cream (4A2) when dry, 2 per mm at base, up to 2 mm long when dry. Context translucent when fresh. Stipe translucent when fresh, up to 12 mm long and 3 mm in diam when dry.

##### Hyphal structure.

Hyphal system monomitic; generative hyphae with clamp connections. Contextual hyphae hyaline, thin- to slightly thick-walled, frequently branched, interwoven, 2–6 μm in diam. Tramal hyphae hyaline, thin-walled, frequently branched, interwoven, 3–4 μm in diam. Cystidia fusiform, 19–50 × 2–6 μm; cystidioles absent. Hyphidia occasionally branched. Basidia four-celled, barrel-shaped, subglobose or globose, 10.5–12 × 10–11 μm; sterigmata up to 13 μm long and 2–3 μm in diam. Probasidia fusiform to lageniform, 10–15 × 6–9 μm. Basidiospores ovoid to broadly ellipsoid, hyaline, thin-walled, IKI–, CB–, (6.8–)7–8.5(–9) × (5.2–)5.5–7(–8) μm, *L* = 7.61 μm, *W* = 6.32 μm, *Q* = 1.19–1.22 (60/2).

##### Additional specimens examined (paratypes).

China • Xizang Autonomous Region, Motuo County, on fallen trunk of *Abies*, elev. 3400 m, 20 September 2014, Cui 12214 (BJFC017128); the road from Bomi to Motuo, elev. 3217 m, 22 August 2018, WXH 5603 (HKAS112529).

#### Pseudohydnum rhododendri

Taxon classificationFungiAuricularialesHyaloriaceae

Xin Zhang & Y.C. Dai sp. nov.

F55F2CA5-E0DC-5FCB-84B2-328C3849400C

862557

Fig. 3

##### Diagnosis.

It is characterized by shell-shaped, pileate basidiomata with a rudimentary stipe and pale mouse-gray pileal surface when fresh, barrel-shaped to subglobose basidia, branched hyphidia, and growing on *Rhododendron*.

**Figure 3.**
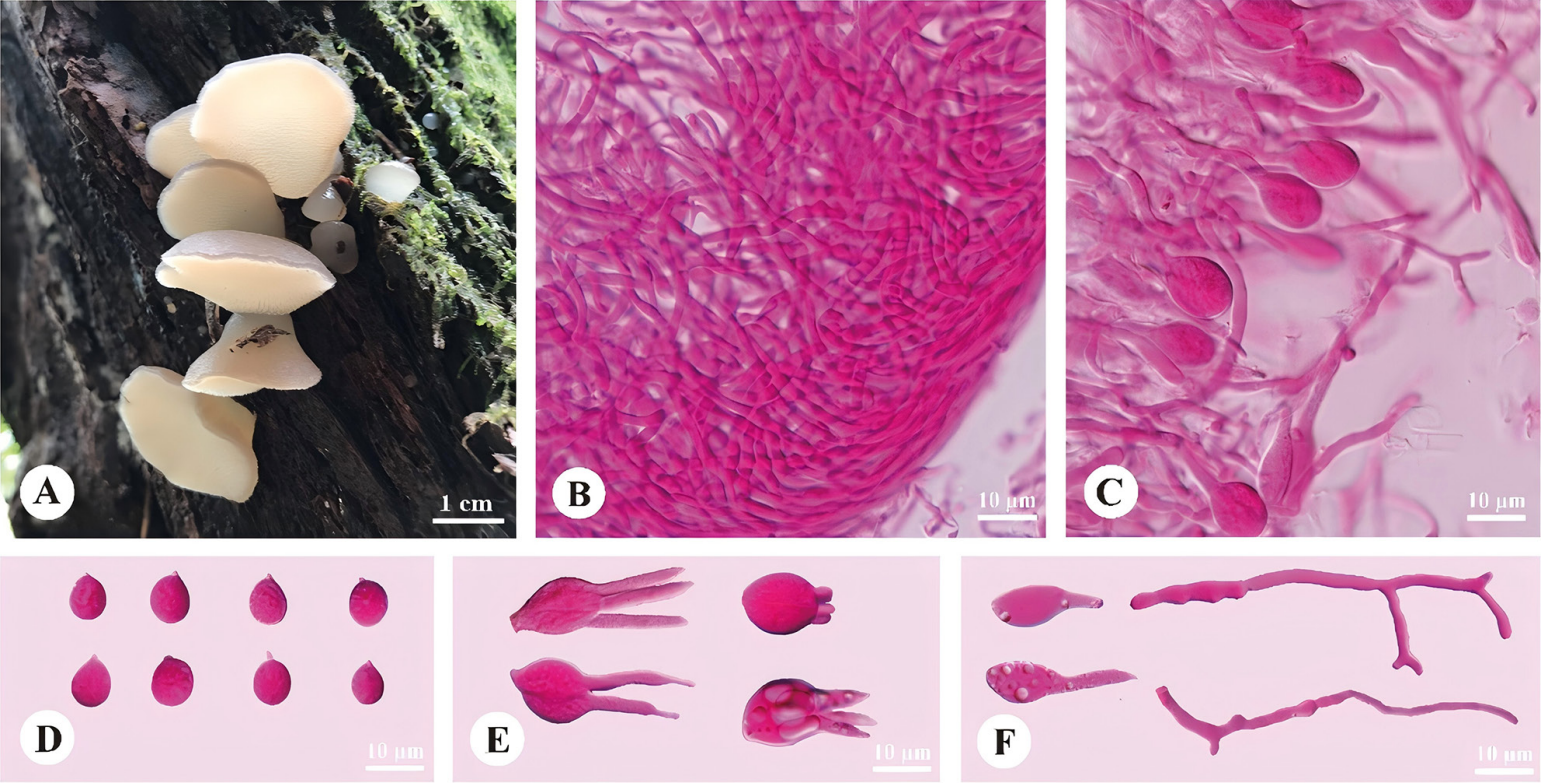
Basidiomata and microscopic structures of *Pseudohydnum rhododendri* (holotype, Cui 14495). **A**. Basidiomata; **B**. Tramal hyphae; **C**. A section of the hymenium; **D**. Basidiospores; **E**. Basidia; **F**. Cystidioles and hyphidia.

##### Holotype.

China • Guangxi Zhuang Autonomous Region, Guilin, Maoershan National Nature Reserve, on living tree of *Rhododendron thunbergii*, elev. 1732 m, 13 July 2017, Cui 14495 (holotype, BJFC029363).

##### Etymology.

*Rhododendri* (Lat.): refers to the species growing on *Rhododendron*.

##### Fruitbody.

Basidiomata annual, pileate with a rudimentary stipe, imbricate, gelatinous when fresh, brittle when dry. Pilei shell-shaped, projecting up to 1.2 cm, 2.1 cm wide and 1 mm thick at base when dry. Pileal surface pale mouse-gray (7C2) when fresh, become chestnut (7E7) when dry. Spines white (1A1) and conical when fresh, become buff (4A4) when dry, 4–6 per mm at base, up to 1 mm long. Context translucent when fresh.

##### Hyphal structure.

Hyphal system monomitic; generative hyphae with clamp connections. Contextual hyphae hyaline, thin-walled, frequently branched, interwoven, 10–18 μm in diam. Tramal hyphae hyaline, thin-walled, occasionally branched, interwoven, 6–8.5 μm in diam. Cystidioles fusiform to lageniform, 19–27 × 6–9 μm. Hyphidia occasionally branched. Basidia two- or four-celled, barrel-shaped, ovoid to subglobose, occasionally with guttules, 12–16 × 9.5–11.5 μm; sterigmata up to 16 μm long and 2–3.5 μm in diam. Basidiospores broadly ellipsoid to ovoid, hyaline, thin-walled, IKI–, CB–, (7–)7.5–9(–9.8) × 6–8(–8.8) μm, *L* = 8.2 μm, *W* = 6.96 μm, *Q* = 1.15–1.21 (60/2).

##### Additional specimen examined (paratype).

China • Guangxi Zhuang Autonomous Region, Guilin, Maoershan National Nature Reserve, elev. 1750 m, on living tree of *Rhododendron thunbergii*, 13 July 2017, Cui 14495 (BJFC029364).

## Discussion

Morphological examination and phylogenetic analysis identified 27 taxa of *Pseudohydnum* as a well-supported monophyletic group (98% ML, 1 BPP; Fig. 1), distributed across Asia, Europe, North and South America, and Oceania. In this study, two new species from Xizang and Guangxi, China, viz., *Pseudohydnum fusiformis* and *P. rhododendri*, are formally described based on morphological and phylogenetic evidence.

*Pseudohydnum fusiformis* was found at high elevations (> 3200 m) in Xizang, southwestern China, growing on fallen trunks of *Abies delavayi* var. *motuoensis*. Phylogenetically, it formed a small subclade with *P. omnipavum* (0.91 BPP; Fig. 1), but *P. omnipavum* differs from *P. fusiformis* in having a finely tomentose to strigose, watery-grayish pileal surface, shorter basidiospores (5.8–7.3 μm vs. 7–8.5 μm in length), and is distributed in North America (Spirin et al. 2023). This subclade belongs to a larger well-supported clade (99% ML, 1 BPP), which also includes another subclade (97% ML, 1 BPP) containing five Eurasian species (*P. alienum*, *P. himalayanum*, *P. laricicola*, *P. sinobisporum*, and *P. translucens*). This larger clade is characterized by species with predominantly pale-colored basidiomata and a frequent association with conifers, especially *Abies* (Zhou et al. 2022, 2023; Spirin et al. 2023; Wang et al. 2025b). Morphologically, *P. fusiformis* is most strikingly characterized by the presence of fusiform cystidia, a feature otherwise known only from *P. cystidiatum* within the genus. However, *P. cystidiatum* differs from *P. fusiformis* in having a rudimentary or sessile stipe, tapering, broader cystidia (15.5–16.7 μm vs. 2–6 μm in width), and growing on decayed wood in subtropical lowland forests of Vietnam and Yunnan, China, whereas *P. fusiformis* grows on *Abies* in high-elevation boreal forests (Spirin et al. 2023). Among the *Abies*-associated species, *P. himalayanum*, *P. abietinum*, and *P. sinogelatinosum* share a similar pinkish pileal surface with *P. fusiformis*, but they differ from *P. fusiformis* by having basidiospores with a large guttule; *P. abietinum* further differs by having a rudimentary stipe, and *P. himalayanum* and *P. sinogelatinosum* differ by having longer basidia (12–17.5 μm in *P. himalayanum* and 12–15 μm in *P. sinogelatinosum* vs. 10.5–12 μm in *P. fusiformis*; Zhou et al. 2022, 2023). Similarly, *P. alienum* and *P. translucens* differ from *P. fusiformis* by having a watery-grayish, strigose pileal surface and denser hymenial spines (6–8 per mm in *P. alienum* and *P. translucens* vs. 2 per mm in *P. fusiformis*; Spirin et al. 2023).

*Pseudohydnum rhododendri* was found in Guangxi, southern China, growing on living *Rhododendron thunbergii* in a montane forest (1732–1750 m elevation). Phylogenetically, it formed an independent lineage (100% ML, 1 BPP; Fig. 1) within *Pseudohydnum*. Morphologically, *P. alienum*, *P. cystidiatum*, *P. orbiculare*, *P. tasmanicum*, and *P. umbrosum* share a generally grayish pileal surface and a rudimentary or absent stipe with *P. rhododendri*. However, *P. alienum*, *P. cystidiatum*, and *P. orbiculare* differ from *P. rhododendri* in spine density (6–8 per mm in *P. alienum*, 3–4 per mm in *P. cystidiatum*, 0.5–1 per mm in *P. orbiculare* vs. 4–6 per mm in *P. rhododendri*) and consistently four-celled basidia (Zhou et al. 2022; Spirin et al. 2023). *P. umbrosum* shares a similar spine density with *P. rhododendri* but differs from *P. rhododendri* in having a dark brown to blackish pileal surface and occurring on conifers in Russia and northeastern China (Spirin et al. 2023). *P. tasmanicum* resembles *P. rhododendri* by having two- to four-celled basidia and almost the same shape and size of basidiospores but differs by having simple hyphidia, sparser hymenial spines (2–3 per mm vs. 4–6 per mm), and growing on *Eucalyptus* and *Nothofagus* in Australia (Zhou et al. 2022). Most significantly, *P. rhododendri* is the only species in the genus that possesses cystidioles and is currently known from living *Rhododendron* in Guangxi, southern China—a highly specialized host association unprecedented in the genus and recorded here for the first time.

In conclusion, the discovery of *Pseudohydnum fusiformis* with fusiform cystidia—a feature previously known only from *P. cystidiatum*—and a high-elevation *Abies* habitat in Xizang and *P. rhododendri* with cystidioles, two- to four-celled basidia, and an unprecedented host association on living *Rhododendron* in Guangxi, southern China, together highlight the morphological and ecological diversity of *Pseudohydnum* in China. The morphological features observed (e.g., basidia type, presence of cystidia, host specificity) suggest that host associations may have played a role in species diversification within *Pseudohydnum*, but further studies are needed to test this hypothesis.

## Supplementary Material

XML Treatment for Pseudohydnum fusiformis

XML Treatment for Pseudohydnum rhododendri

## Additional information

### Artificial Intelligence (AI) use

The authors accept full responsibility for the content of the manuscript, including the disclosure of any use of AI.

No AI tools were used in the preparation of this manuscript.

### Author ORCIDs

X. Zhang https://orcid.org/0009-0005-8363-7852

H.-M. Zhou https://orcid.org/0000-0002-0724-5815

Z.-H. Tu https://orcid.org/0009-0009-8444-9270

G.-Y. Zeng https://orcid.org/0009-0009-8609-1612

Y.-C. Dai https://orcid.org/0000-0002-6523-0320

### Data availability

All of the data that support the findings of this study are available in the main text.
